# So many options but one choice: the human body prefers α-tocopherol. A matter of stereochemistry.

**Published:** 2008-11-15

**Authors:** Manolescu B, Atanasiu V, Cercasov C, Stoian I, Oprea E, Buşu C

**Affiliations:** * Department of Biochemistry, Faculty of Medicine, University of Medicine and Pharmacy “Carol Davila”; ** Department of Organic Chemistry, Faculty of Chemistry, University of Bucharest

## Abstract

α-Tocopherol belongs to the group of vitamin E vitamers. Recent years findings indicate that α-tocopherol is more than just a simple fat-soluble anti-oxidant as it was found that it can also regulate gene expression. From all vitamin E vitamers human body preferentially retains α-tocopherol, but the reasons for this preference are still elusive. Different studies indicated that human body, through the action of two hepatic proteins, α-tocopherol transfer protein (α-TTP) and cytochrome P450 4F2 (CYP4F2), is able to make subtle structural differences between different vitamin E forms. This is an example of stereochemistry used as a discrimination factor between molecules with different biological activities.

## 1. Introduction

Vitamin E is the generic name used to designate eight chemically related compounds which differ in the number and positions of methyl groups on the chromanol ring and in the saturation and stereochemistry of the phytyl tail. Human body is able to concentrate and retain from food sources of α-tocopherol. This is accomplished through the action of two hepatic proteins α-TTP and CYP4F2, respectively. α-TTP acts as a vitamin E retention factor, whereas selectivity among the different vitamin E vitamers is driven by the substrate specificity of CYP4F2, which catalyzes the first step in vitamin E metabolism.

## 2. Vitamin E structure and functions

All vitamin E vitamers have a chromanol ring with different extents of substitution. There are four tocopherols designated α-,β-,γ-, and δ-tocopherol and their four corresponding tocotrienols.

**Fig. 1:**
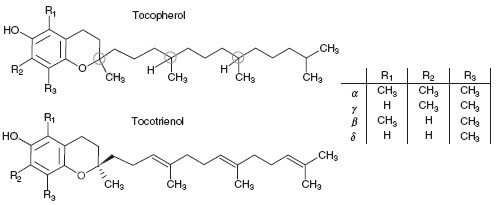
Chemical structures of tocopherols and tocotrienols. The circles mark the three chiral centers in tocopherols (2, 4’, 8’).

The tocopherols have a phytyl tail inserted on the chromanol ring and differ in respect to their methylation degree [**1**]. The tocotrienols have an unsaturated tail with three double bonds two of which have *trans* configuration. Each one of the four tocopherols has three chiral centers, represented by carbon atoms 2, 4’ and 8’ respectively. As a consequence of this for each tocopherol there are eight possible stereoisomers (*RRR, RSR, RRS, RSS, SRR, SSR, SRS, and SSS*). RRR-α-tocopherol is the naturally occurring form of α-tocopherol. It was found that natural tocopherols have the R configuration at all chiral centers and that stereoisomers with R configuration at C2 are more biologically active that those with S configuration [**2**,**3**]. The synthetic vitamin E is called *all-rac-α-*tocopherol and consists of a mixture of equal amounts of the eight possible stereoisomers (rac=racemic).

Vitamin E functions can be divided into anti-oxidant (free radical chain-breaking antioxidant) and non-antioxidant (mediated through protein kinase C and tocopherol-associated proteins and tocopherol-binding proteins) [**4**,**5**].

Vitamin E, alone and in conjunction with other compounds (vitamin C, polyphenols, and selenium), is one of the components of the first line of defense against lipid peroxidation. α-Tocopherol acts as a chain-breaking antioxidant preventing thus auto-oxidation of polyunsaturated fatty acids (PUFAs) from plasma membrane phospholipids and from lipoprotein particles. Peroxyl radicals (ROO•) tend to react faster with α-tocopherol (vit. E-OH) than with hydrogen atoms from bis-allylic positions within PUFAs [**6**]. 

**Figure:**
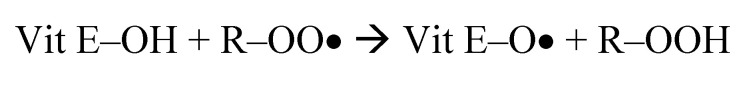


**Fig. 2:**
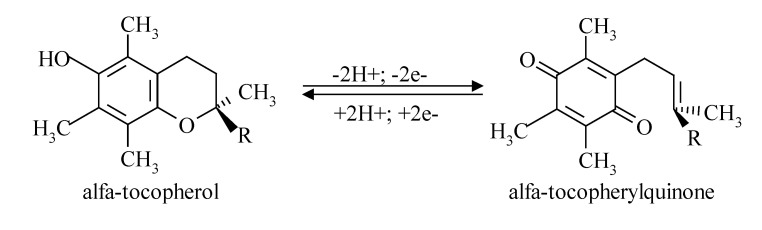
α-Tocopherol acting as a chain-breaking anti-oxidant.

The main factor that enables α-tocopherol to act as a chain-breaking anti-oxidant is represented by its ability to reversible interconvert between α-tocopherol and α-tocopherolquinone forms (**Figure 2**).

α-Tocopherol will be regenerated from the α-tocopheroxyl radical (vit E–O•) in a subsequent reaction with a reducing agent such as vitamin C or glutathione [**7**-**9**]. 

**Figure:**
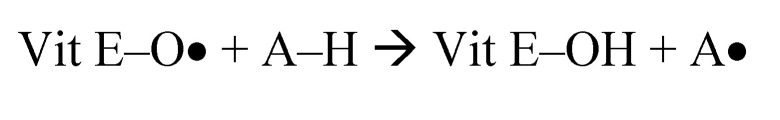


Recent data suggest that α-tocopherol can inhibit free radical generation in a manner independent of its „scavenger radical” properties. α-Tocopherol is able to limit superoxide production through inhibition of NADPH oxidase activity [**10**]. Also, α-tocopherol can inhibit phospholipase A2 and cyclooxygenase activities with a concomitant increase in α-glutamylcysteinil synthetase activity resulting in an overall diminution of free radicals production [**11**-**13**].

There are many studies to indicate that α-tocopherol influences gene expression independent of its anti-oxidant functions (**Table I**).

**Table 1:** Effects of α-tocopherol upon expression of genes.

Up-regulated genes	OBSERVATIONS
• α-tocopherol transfer protein (α-TTP) gene,	α-TTP is responsible for the specific uptake of α-tocopherol and for the control of α-tocopherol plasma level
• CYP3A4 and CYP3A5 genes	Involved in liver tocopherols catabolism [**14**]
• SR-B1 scavenger receptor gene	α-tocopherol-depleted rats show increased expression of scavenger receptor SR-B1 [**15**]
• α-tropomyosin gene	Extracellular protein [**16**]
• Connective tissue growth factor (CTGF) gene	α-tocopherol induces CTGF gene expression 1,8 fold [**17**]
Down-regulated genes	
• CD36 scavenger receptor gene	At physiological concentration α-tocopherol down-regulates CD36 mRNA transcription and protein expression [**18**]
• SR-A scavenger receptor gene	The same as for CD36 scavenger receptor [**19**]
• Collagenase (MMP-1) gene	Extracellular protein [**20**]
• Collagen α1(I) gene	α-Tocopherol induces the decrease of liver collagen mRNA [**21**]

## 3. Vitamin E metabolism

All vitamin E vitamers are absorbed with very similar rates. It is speculated that tocotrienols are absorbed better than the corresponding tocopherols [**22**]. Being a fat-soluble compound, vitamin E absorption depends upon bile acids and pancreatic secretion in order to form micelles for uptake by entherocytes. Inside the entherocytes, tocopherols and tocotrienols are assembled together with triacilglycerols, cholesterol, phospholipids, carotenoids and apolipoprotein B48 into chylomicrons [**23**]. There is no discrimination between different vitamin E vitamers during intestinal absorption [**24**,**25**].

In the circulation, chylomicrons undergo triacilglycerols lipolysis by lipoprotein lipase. There are some studies suggesting the fact that lipoprotein lipase is able to deliver vitamin E vitamers to different cells [**26**,**27**]. Some of the newly absorbed vitamin E is transferred to other circulating lipoproteins, while the rest remains in the composition of chylomicron remnants. The exchange of vitamin E vitamers between different plasma lipoprotein particles is catalyzed by the phospholipid transfer protein (PLTP) [**28**,**29**].

The liver takes up chylomicron remnants and secretes VLDL into circulation. Studies using deuterated tocopherols indicated that RRR-α-tocopherol is preferentially secreted by hepatocytes. There is evidence that α-tocopherol becomes associated with VLDL after its secretion, probably in the sinusoidal space [**30**]. VLDL particles are not essential for tissue distribution of α-tocopherol, because in mice lacking VLDL the α-tocopherol content of HDL significantly increases [**31**]. HDL particles are also involved in reverse transport of α-tocopherol from peripheral tissues back to the liver. They also deliver α-tocopherol to different tissues. The transfer of α-tocopherol from HDL particles to tissues involves a pathway related to SR-B1 [**32**].

Unlike other fat-soluble vitamins, vitamin E is not stored. The metabolism of vitamin E vitamers consists of an initial ω-hydroxylation. The hydroxyl group will be oxidized to a carboxyl group. The final step is represented by a sequence of β-oxidation leading to α-, β-, γ-, and δ-CEHC (2’-carboxyethyl-6-hydroxychromane). The same pathway is followed by the four tocotrienols. The initial ω-hydroxylation is carried out by CYP4F2 and probably CYP3A4 [**33**,**34**]. Prior to excretion in either the bile or the urine, CEHCs are sulfated or glucuronidated [**35**]. There is evidence that unmodified α-tocopherol is excreted into bile, a process which depends upon two ATP-binding cassette (ABC) transporters located in the canalicular membranes of hepatocytes (MDR1 and MDR3) [**36**]. It is speculated that MDR1 is involved in α-tocopherol bile excretion under conditions of high-dose supplementation [**36**]. 

## 4. Secretion of α-tocopherol by the liver 

There is a disagreement between diet and plasma concentrations of α-tocopherol and γ-tocopherol. A typical US diet contains large amounts of soybean oil which is rich in γ-tocopherol (approximately 70mg), but scarce α-tocopherol (approximately 7mg) [**38**]. Plasma α-tocopherol concentrations in human range from 11-37μmol/L, while γ-tocopherol concentrations are roughly 2-5μmol/L [**39**]. This paradox is a consequence of (1) the selectivity of the hepatic α-TTP and (2) the regulation of vitamin E hepatic metabolism and excretion.

**4.1. α-Tocopherol transfer protein selectively binds α-tocopherol**

The central factor that regulates α-tocopherol concentration is α-TTP. α-TTP is a cytosolic protein with a molecular weight of 32kDa coded by a gene located on chromosome 8 (8q13.1-13-3) [**40**]. α-TTP belongs to the CRAL-TRIO family along with the cellular retinaldehyde binding protein (CRALBP), yeast phosphatidylinositol transfer protein (Sec14p), and supernatant protein factor (SPF) involved in cholesterol biosynthesis [**41**]. α-TTP was identified in hepatocytes, human brain and human placenta [**23**]. The affinities of α-TTP for various vitamin E vitamers and the biological activities of α-tocopherol stereoisomers are presented in **Table II** and **III**.

**Table 2:** Affinities of α-TTP for vitamin E vitamers [**42**].

RRR-α-tocopherol	100%
β-tocopherol	38%
γ-tocopherol	9%
δ-tocopherol	2%
α-tocopherol acetate	2%
α-tocopherol quinone	2%
SRR-α-tocopherol	11%
α-tocotrienol	12%
trolox	9%

**Table 3:** Biological activities of α-tocopherol stereoisomers [**3**].

RRR-α-tocopheryl acetate	100%
RRS-α-tocopherol	90%
RSS-α-tocopherol	73%
SSS-α-tocopherol	60%
RSR-α-tocopherol	57%
SRS-α-tocopherol	37%
SRR-α-tocopherol	31%
SSR-α-tocopherol	21%

Crystallographic studies revealed that α-tocopherol is bounded by α-TTP inside a hydrophobic pocket through van der Waals contacts [**43**,**44**]. Inside the hydrophobic pocket there are also four water molecules: two are hydrogen-bonded to the hydroxyl group of the chromanol ring, one is hydrogen-bonded to the oxygen atoms of Val182 and Leu189, and the fourth water molecule is hydrogen-bonded to the hydroxyl group of the Ser140 [**44**]. 

Inside the hydrophobic pocket of α-TTP there is an indent generated by the side chains of Phe133, Val182 and Ile179. It plays an important role in discrimination among different stereoisomers of α-tocopherol as it can accommodate only the chiral C2 with R configuration [**43**]. 

The factors responsible for ligand discrimination of α-TTP are (1) the methylation degree of the chromanol ring, (2) the presence of the phytyl tail, and (3) the R configuration at carbon 2 where the phytyl tail attaches to the chromanol ring [**41**]. The extreme low affinity of α-TTP for tocotrienols is explained by the presence of the three double bonds with rigid configurations, which impede the unsaturated tail to accommodate in the hydrophobic pocket of the protein.

Inside the hepatocytes, α-TTP aquires α-tocopherol from endosomes and then moves to plasma membrane where α-tocopherol is released. Then, α-tocopherol can be incorporated into nascent VLDL particles.

**4.2. Regulation of hepatic vitamin E metabolism and excretion**

Hepatic metabolism and excretion of vitamin E vitamers is the second regulatory level by which human body selectively retains RRR-α-tocopherol.

The first step of the hepatic vitamin E metabolism is represented by the CYP4F2 ω-hydroxylation. CYP4F2 is more active toward γ-tocopherol than toward α-tocopherol [**45**]. It was found that the critical determinants of the rate of ω-hydroxylation are (1) the position of methyl groups on the chromanol ring, particularly at C5, and (2) unsaturation of the side chain [**46**]. The presence of a methyl group at C5 leads to decrease susceptibility of ω-hydroxylation. It was also found that CYP4F2 has allosteric properties as α-tocopherol acts as a positive allosteric effector that stimulates the ω-hydroxylation rate of other vitamin E vitamers.

## 5. Conclusions

There are several gaps in the knowledge about the regulation of vitamin E concentration. All performed studies indicate that there is a high preference for α-tocopherol, but the exact reason of this preference is still controversial and remains undetermined. Acting in conjunction, two hepatic proteins, α-TTP and CYP4F2 are greatly responsible for the selective retention by the human body of α-tocopherol. Further studies are needed to obtain a clearer image about the regulation of α-tocopherol metabolism.
